# Real-time numerical system convertor *via* two-dimensional WS_2_-based memristive device

**DOI:** 10.3389/fncom.2022.1015945

**Published:** 2022-09-14

**Authors:** Xing Xin, Liyao Sun, Jiamei Chen, Youzhe Bao, Ye Tao, Ya Lin, Jingyao Bian, Zhongqiang Wang, Xiaoning Zhao, Haiyang Xu, Yichun Liu

**Affiliations:** Center for Advanced Optoelectronic Functional Materials Research, Key Laboratory of UV-Emitting Materials and Technology, Ministry of Education, Northeast Normal University, Changchun, China

**Keywords:** memristor, numerical system convertor, oxygen plasma, transition metal dichalcogenides, tungsten disulfide

## Abstract

The intriguing properties of two-dimensional (2D) transition metal dichalcogenides (TMDCs) enable the exploration of new electronic device architectures, particularly the emerging memristive devices for in-memory computing applications. Implementation of arithmetic logic operations taking advantage of the non-linear characteristics of memristor can significantly improve the energy efficiency and simplify the complexity of peripheral circuits. Herein, we demonstrate an arithmetic logic unit function using a lateral volatile memristor based on layered 2D tungsten disulfide (WS_2_) materials and some combinational logic circuits. Removable oxygen ions were introduced into WS_2_ materials through oxygen plasma treatment process. The resistive switching of the memristive device caused by the thermophoresis-assisted oxygen ions migration has also been revealed. Based on the characteristics of excitatory postsynaptic current (EPSC), paired-pulse facilitation (PPF), and spike rate dependent plasticity (SRDP), a real-time numerical system convertor was successfully accomplished, which is a significant computing function of arithmetic logic unit. This work paves a new way for developing 2D memristive devices for future arithmetic logic applications.

## Introduction

The explosive increase of data along with the rapid development of big data analytics and internet of things demands innovative device architectures and alternative materials (Li et al., 2018; Dai et al., 2019; Zhu et al., 2019). In-memory computing, which can process and store information simultaneously, has the potential to address the critical challenge affecting current computing platforms (von Neumann computer architecture) (Ielmini and Wong, 2018; Jain et al., 2018; Aqib et al., 2019; Verma et al., 2019). Memristor, possessing the characteristics of simple structure, low power consumption and non-linear conductance variation, can be employed to accomplish some interesting computing functions, such as arithmetical computing, logical operation, and reservoir computing application (Hu et al., 2018; Mao et al., 2019; Choi et al., 2020; Lin et al., 2020; Zhong et al., 2021). Introducing these memristive devices will not only greatly simplify the peripheral circuit complexity, but also effectively improve the energy consumption and back-end integration potential (Kumar et al., 2022). A more advanced computing function of arithmetic logic unit (ALU) has also been proposed to further improve the development of in-memory computing (Cheng et al., 2019).

Two-dimensional (2D) materials, such as transition metal dichalcogenides (TMDCs), have attracted numerous attention and accelerated the progress of memristive devices in both the storage and computing applications in recent years (Chia and Pumera, 2018; Akinwande et al., 2019; Li et al., 2019; Liu et al., 2019, 2020; Yang et al., 2019; Chen et al., 2020; Wang et al., 2020; Zavabeti et al., 2020; Zhou et al., 2021; Kwon et al., 2022). Wang et al. (2018) have reported a robust layered MoS_2–x_O_*x–*_based memristor through the oxidation of MoS_2_ films in ambient air. The obtained device exhibited an excellent switching performance with an endurance of ∼10^7^ and an ultra-high operating temperature of 340°C (Wang et al., 2018). Lin et al. have used a self-oxidized method to fabricate a stable unipolar switching MoS_2_/graphene/HfSe_2–x_O_*x*_ memristor (Yin et al., 2020). It possessed the ability for the multi-bit data storage and could also be acted as a memory latch or logic gate. In addition, versatile memristive behaviors and functions with dual-mode operation (electrical and optical stimuli) could also be realized, according to the outstanding photoelectrical characteristics of 2D materials. A dynamic memristive device based on 2D tin sulfide (SnS_2_) materials has been built by Sun et al. (2021) demonstrating the optoelectronic reservoir computing for language learning function. It is obvious to see that adjusting the micro-structure of 2D materials is an efficient way to control the properties of memristive devices based on them, which would thereby further widen their application range.

In addition, the implementation of arithmetic logic functions within the memristors is one of the most significant extended applications for in-memory computing. Cheng et al. (2019) have demonstrated several arithmetic logic unit functions through a memristive crossbar, including non-volatile Boolean logic and arithmetic computing, which improves the development of in-memristor computing. In fact, the numerical system convertor as another kind of arithmetic logic operations, which contains binary-to-decimal, binary-to-hexadecimal and decimal-to-hexadecimal types et al., was also usually executed in the arithmetic logic unit (ALU) (Sharma and Kumre, 2020). However, the investigations about the achievement of numerical system convertors through a memristor-based computing architectures have been rarely reported.

WS_2_, a typical semiconductor material with tunable band gap (1.2∼2.1 eV) from multilayer to monolayer (Kuc et al., 2011), possesses high phonon limited electron mobility (Zhang et al., 2014), on/off current ratio (Yue et al., 2018) and light effective mass (Liu et al., 2011), which enable it to apply to low-power memristors as active layer (Luo et al., 2020). Especially, the existence of sulfur vacancies in WS_2_ will introduce localized donor states inside the bandgap, which largely changes its carrier mobility and memristive behavior. Herein, we will demonstrate a lateral memristor based on oxygen-plasma-treated 2D tungsten disulfide (WS_2_) materials. It is worth emphasizing that the corresponding memristive behaviors can be continuously regulated by changing the duration time of oxygen plasma treatment. Because of a lower migration barrier for oxygen ions than that for sulfur ions (Wang et al., 2018), the doped oxygen ions could thermophoresis-assisted migrate under an electric field. Some synapse-based electrical characteristics, excitatory postsynaptic current (EPSC), paired-pulse facilitation (PPF), and spike rate dependent plasticity (SRDP) have been efficiently emulated. In addition, a real-time numerical system convertor was also successfully accomplished, according to the unique accumulation and relaxation characteristics of the 2D WS_2_ materials-based memristor. It has not been demonstrated with 2D TMDCs memristor before and these results will provide a potential approach for the development of future arithmetic logic unit.

## Materials and methods

### Device fabrication

**FIGURE 1:**
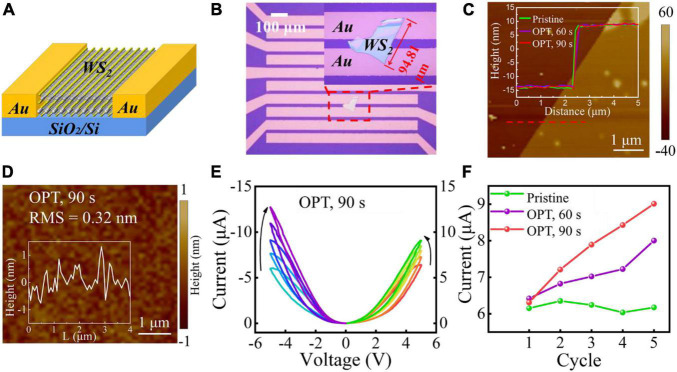
**(A)** A schematic diagram of the lateral Au/WS_2_/Au device. **(B)** A microscopic image of the lateral memristive device with interdigital electrodes. **(C)** The AFM height topography measured at the edge of WS_2_ flake. The inset shows the height profiles measured along the red dotted line in panel **(D)**, illustrating the thickness variation of pristine WS_2_ and oxygen-plasma-treated WS_2_. **(D)** AFM morphology of the layered WS_2_ experiencing OPT for 90 s. The root-mean-square (RMS) roughness value is also presented. The inset is the representative height line scan. **(E)** Typical I-V curves of the memristive device after OPT for 90 s. **(F)** Variation trend of the maximal current value with continuous 5 cycles of pristine devices and after OPT for 60 and 90 s, respectively.

Firstly, the Au electrode with interdigital structure was fabricated through the technology of standard lithography and thermal evaporation. Subsequently, the layered 2D WS_2_ was mechanically exfoliated with the assistance of accurate two-dimensional transfer platform (METATEST E1-T). The schematic diagram of device structure is shown in Figure 1A. In order to introduce the sulfur vacancies and oxygen elements into the layered WS_2_ without completely damaging the material micro-structure, a soft oxygen plasma treatment was conducted under a power of 30 W with variable treatment durations (<90 s).

### Characterization and electrical measurements

The AFM measurements were performed in air using a Bruker Dimension Icon atomic force microscope (Bruker, Germany). The X-ray photoelectron spectroscopy (XPS) was performed on an ESCA-LAB 250 photoelectron spectrometer equipped with X-ray source in Al k α (1486.7 eV). The atomic structure of high-quality single-crystal WS_2_ domain was carried out by TEM on an FEI Titan Themis Cube with an X-FEG electron gun operating at 100 kV. Raman spectra was recorded with a JY HR-800 LabRam Infinity Spectrophotometer with a 488 nm semiconductor laser. The X-ray diffraction (XRD) measurements were conducted using Dmax-2500X (Rigaku, Japan). All the electrical measurements were performed with a source meter (2636A, Keithley), an arbitrary function generator (3390, Keithley), and an oscilloscope (TDS 2012B, Tektronix), and the testing environment was maintained under temperature of ∼25°C and relative humidity of ∼30%.

## Results and discussion

The schematic diagram of device structure and the interdigital electrode structure fabricated through standard lift-off lithography technology are shown in Figures 1A,B. This structure makes the WS_2_ multilayers and electrodes to form a lateral Au/WS_2_/Au memristive device with a channel width of ∼20 μm. For adjusting the micro-structure of the mechanically exfoliated multilayer WS_2_ flakes, the lateral memristive device was treated by a soft oxygen plasma. It was implemented by a source located far from the sample with a low power (30 W) and short exposure time (<90 s). The variation of the micro-structure, physical and chemical properties of WS_2_ induced by the different oxygen plasma exposure times was investigated thoroughly. As shown in Figure 1C, the thicknesses (∼22.5 nm) of multilayer WS_2_ changed a little after the oxygen plasma treatment (OPT), which were measured at the flake edge by atomic force microscope (AFM). Figure 1D and Supplementary Figure 1 show the surface morphology of pristine WS_2_ and WS_2_ flakes treated by oxygen plasma for 60 and 90 s. The corresponding root-mean-square (RMS) roughness values are 0.15, 0.23, and 0.32 nm, respectively. The roughness of WS_2_ flake treated by oxygen plasma for 90 s increased slightly (0.17 nm), compared with that of pristine WS_2_. This phenomenon is consistent with the unobvious variation of the WS_2_ thickness after oxygen plasma treatment in Figure 1C. The representative line scans extracted from the AFM maps (Insets in Figure 1D and Supplementary Figure 1) show that the peak-to-valley height excursion (HE) increases with prolonging oxygen plasma exposure time. The HE value is less than the thickness of monolayer WS_2_ (∼0.7 nm) for 60 s treatment. When exposure time was up to 90 s, the HE value increased to ∼1 nm. It means that maybe a small amount of structural damage was introduced by a long-time OPT. As reported, when performing energetic plasma treatments, the exposed multilayer TMDCs may undergo a significant etching (Chen et al., 2013; Giannazzo et al., 2017). To forbid a more serious damage of WS_2_, oxygen plasma exposure time was controlled within 90 s in our experiments. Figure 1E shows the typical I-V curves of the lateral Au/WS_2_/Au memristive device after OPT for 90 s, from which we can see that the treated memristor shows an analogy memristive behavior. The current amplitudes would both increase along with either positive or negative DC voltage sweeping operations, which could result from the same Au noble electrodes. In contrast, as shown in Figure 1F and Supplementary Figure 2, there exists no obvious memristive behavior although continuous DC voltage sweep was operated on both the pristine and 60 s-OPT devices. The related physical mechanism will be discussed as follows.

**FIGURE 2:**
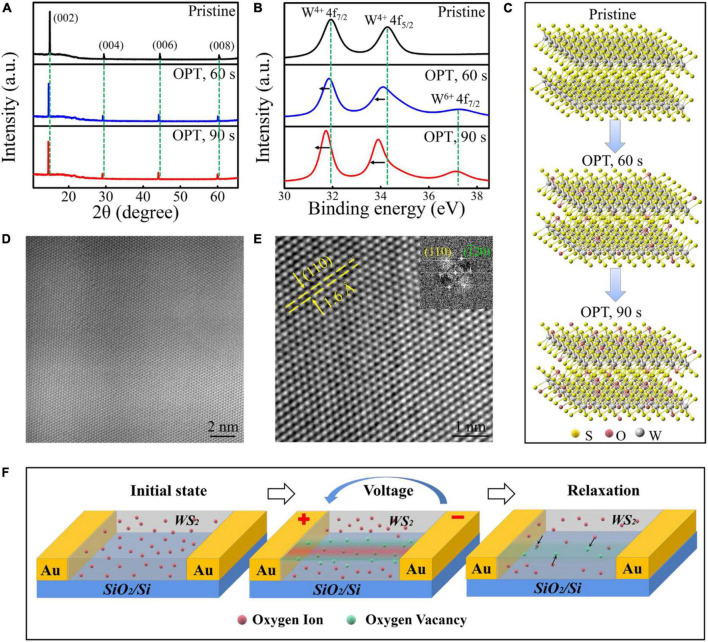
**(A)** XRD and **(B)** XPS spectra of WS_2_ before and after oxygen plasma treatment for 60 and 90 s. **(C)** The structural schematic diagram for the variation of multilayer WS_2_ with different oxygen plasma exposure times. HRTEM images at **(D)** low and **(E)** high magnification performed at multilayer WS_2_ interior after oxygen plasma treatment for 90 s. The inset in panel **(E)** is the corresponding FFT image. **(F)** A schematic diagram for the memristive mechanism of the lateral memristive device.

For investigating thoroughly, the effect on the memristive characteristic of the lateral Au/WS_2_/Au device from oxygen plasma treatment, the X-ray diffraction (XRD) measurements were conducted as shown in Figure 2A. There are four main peaks for pristine multilayer WS_2_, which locate at 14.4° (002), 28.9° (004), 44.0° (006), and 59.9° (008), respectively. After OPT for 60 and 90 s, every WS_2_ peak has a left shift compared to pristine WS_2_ peaks. These results illustrate that the WS_2_ crystal structure was unchanged after OPT. Instead, it proves that oxygen plasma will introduce oxygen doping into WS_2_, which induced the increase of lattice spacing and the left shift of WS_2_ peaks. The Raman spectra in Supplementary Figure 3 also present the same conclusion. Then, we conducted the X-ray photoelectron spectroscopy (XPS) measurements to further confirm the successful oxygen doping of WS_2_ by oxygen plasma (Figure 2B). It has been reported that some oxygen ions may also be injected to dope the sulfur vacancies (Esfandiari and Mohajerzadeh, 2019; Esfandiari et al., 2020). It is worth noting that the peak at 37.2 eV after OPT corresponds to the binding energy of W^6+^, indicating the oxidation and doping of WS_2_. The binding energy of W^4+^ of pristine WS_2_ decrease after oxygen plasma, suggesting the electron transfer from W^4+^ to W^6+^. We have used a schematic diagram to intuitively exhibit the structure evolution with different oxygen plasma exposure times (Figure 2C). Figures 2D,E are the high-resolution transmission electron microscopy (HRTEM) images detected at WS_2_ flake after oxygen plasma treatment for 90 s. The distance of the (110) plane with the value of 1.6 Å was identified by a fast Fourier transform (FFT) image as shown in the inset of Figure 2E, which was in good agreement with its theoretical value. The contrast in HRTEM image is related to the stacking of columns of S and W atoms in multilayer WS_2_. Due to the low atomic numbers of oxygen, O atoms in WS_2_ are invisible. However, the results show the WS_2_ lattice was slightly affected by the soft OPT. Figure 2F shows the memristive mechanism for the lateral memristive device. The oxygen ions distribute uniformly in the surface of 2D WS_2_ materials after the oxygen plasma treatment. Comparing with the inherent sulfur ions, the doped oxygen ions possess a lower migration barrier (Wang et al., 2018). Therefore, under an electric field, the oxygen ions could gradually migrate toward the anode since thermophoresis effect would dominate due to the steep radial temperature gradient produced by Joule heating (Wang et al., 2018). The resistive switching mechanism primarily based on the high conductive oxygen vacancies region evolution. In addition, the short time relaxation process after removing the voltage pulse maybe caused by the recombination of oxygen ions around oxygen vacancies region.

**FIGURE 3:**
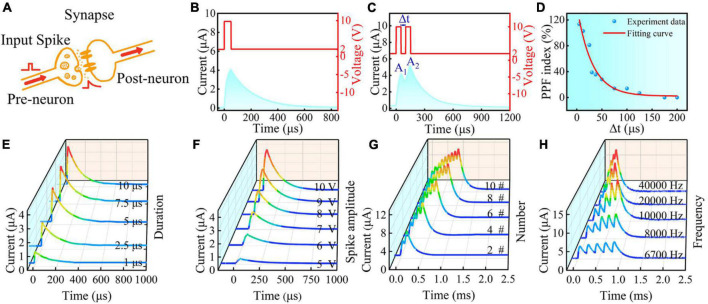
**(A)** Illustrations of the biological synapse based on the WS_2_ memristor after OPT for 90 s. Current response of the lateral Au/WS_2_/Au memristor under **(B)** a single spike and **(C)** paired spikes. **(D)** PPF index versus the relative spike timing. Current response under a single spike with **(E)** different duration times and **(F)** amplitude of input spikes. Current response under **(G)** different spike numbers and **(H)** different frequencies.

Such gradual change behavior of conductance in the lateral Au/WS_2_/Au memristive device is just appropriate for emulating the synaptic learning functions. As shown in Figure 3A, the synaptic weight could be continuously adjusted through various input spikes. The current response of this memristor under a single spike (10 V, 50 μs) was shown in Figure 3B. We can see that the square pulse triggered an abrupt increase of current signal, and then subsequently recovered to the initial state. Such characteristic is very similar to the excitatory postsynaptic current (EPSC) phenomenon of biological synapse. Interestingly, the relaxation process of current signal provides the basis for achieving temporal correlation between two neighboring spikes. One typical temporal correlation between spikes is the paired-pulse facilitation (PPF) function, which is demonstrated in Figure 3C. Apparently, the EPSC value after the second spike is larger than that of the first one, which exactly reflects the PPF effect of the biological synapse. The PPF effect becomes weaker with increasing interval time, as presented by the PPF index in Figure 3D. The current response of the memristor under different single spike and multiple spikes was also investigated. Figures 3E,F show the current variation with different duration times and amplitude of input spikes, from which we can see that a longer or larger input spike will trigger a higher current signal. However, the relaxation time after different stimulus intensity did not show a significant variation. Correspondingly, the peak value of the EPSC shows an obvious increase with more spike numbers and higher frequencies (Figures 3G,H), which can be used to realize the high-pass frequency filter. These essential electrical properties provide the basis for the following numerical system convertor application.

**FIGURE 4:**
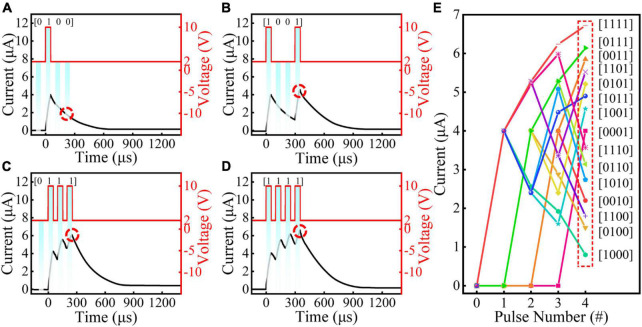
**(A–D)** The current response of the lateral Au/WS_2_/Au memristive device to 4 different pulse streams: [0100], [1001], [0111] and [1111], respectively. **(E)** 16 peak current distribution of the memristive device with 16 kinds of 4 bits binary coding.

Taking advantage of the current response of the lateral Au/WS_2_/Au memristive device to different stimulus intensity, especially to the spiking number and frequency, we have conducted the response experiments of a series of 4 bits binary coding. As shown in Figures 4A–D, extensive tests were carried out to characterize the memristor response to different temporal inputs. We can see that the current response processes to different binary coding ([0100], [1001], [0111], and [1111]) are unique. Thus, it is expected that the read current (immediately after the pulse train), can also be well distinguished between the different binary coding. We have further collected 16 current response results for all 4 bits binary coding to verify this speculation (Figure 4E). This interesting gradual accumulation and relaxation behaviors can also be used for the reservoir computing application.

**FIGURE 5:**
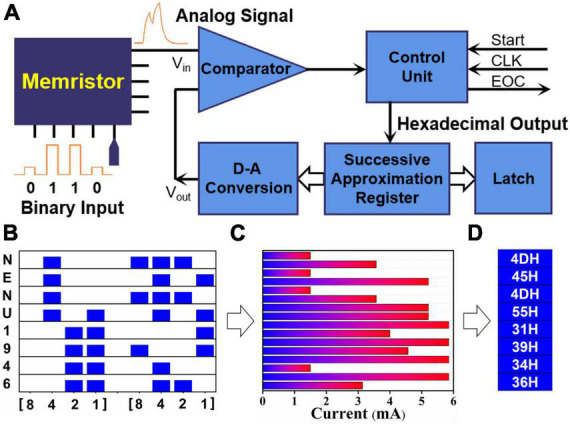
**(A)** The numerical system convertor circuit combining the memristive device and some logic gate circuits. **(B)** The input ASCII code of a string of letters of “NENU1946.” **(C)** The collected peak value of the analog signal output from the memristive device. **(D)** The binary to hexadecimal transformation results from the input ASCII codes.

The up-to-date computers are all based on binary computing and storage systems. Numerical system convertor is a significant computational function of arithmetic logic unit (ALU) of central processing unit (CPU). Especially, the transformation between binary and hexadecimal is one of the most commonly used numerical convertor process, such as the compilation process of assembler source program. That is, the numerical system convertor process needs to occupy the system bus of computer system. Therefore, it is extremely willing to implement the numerical system convertor *via* several electron devices or logic gate circuits only. Herein, according to the current response characteristics of our lateral Au/WS_2_/Au memristive device, the numerical convertor operation from binary to hexadecimal has been realized. As shown in Figure 5A, we have designed the numerical convertor circuit with some basic function modules. A 4-bit binary signal, which can be regarded as the input signal (V_*in*_) of the numerical convertor circuit, was firstly imported into the memristive device. Then, an analog signal outputs from the other electrode. The numerical converting operation was activated by the start signal. The highest order of the successive approximation register is set to “1” (high level), and all the others are set to “0” (low level). At this point, the output voltage (V_*out*_) is half of the full range. Through the comparison process, the highest order was maintained as “1” if the V_*out*_ signal was smaller than the V_*in*_ signal, which is the maximum value of the output signal of the memristive device, and vice versa. Once the V_*in*_ signal is smaller than that of the full range, the second high order will be set to “1.” It will be judged if the V_*in*_ signal is larger than quarter of the full range. The hexadecimal number can be confirmed by only 4 times recurrence. After a series of comparison operation, the control circuit will output the signal of End of Conversion (EOC). Subsequently, the final contents in the successive approximation register will be transmitted into the latch as the result of numerical converting operation. As shown in Figure 5B, a string of letters of “NENU1946” were programmed into our lateral Au/WS_2_/Au memristive device in the form of American standard code for information interchange (ASCII) code. Through extracting the peak value of the output analog signal, the maximal current value was collected as shown in Figure 5C. The hexadecimal number of the letters of “NENU1946,” which is 4DH, 45H, 4DH, 55H, 31H, 39H, 34H, and 35H, are successfully converted in Figure 5D. The above results demonstrate that the numerical system convertor could be realized through the 2D memristive device integrated with several logic gate circuits.

## Conclusion

In summary, a binary-to-hexadecimal numerical system convertor was successfully achieved based on the especial current response characteristics of the lateral Au/WS_2_/Au memristive device. The 2D WS_2_ based memristive device was fabricated through standard lift-off lithography and mechanically exfoliation technologies. The memristive characteristics of our lateral device could be continuously regulated with oxygen plasma treatment time. Several essential synaptic functions, such as EPSC, PPF and SRDP were demonstrated in the memristive device. In addition, this 2D WS_2_ based device could be used in numerical system convertor application only integrating with some combinational logic circuits. This work provides a feasible idea for enabling 2D memristor to accomplish the arithmetic application, which will further broaden the scope for in-memory computing.

## Data availability statement

The original contributions presented in this study are included in the article/Supplementary material, further inquiries can be directed to the corresponding authors.

## Author contributions

All authors listed have made a substantial, direct, and intellectual contribution to the work, and approved it for publication.
