# Supplementary material for: Real-time numerical system convertor via two-dimensional WS2-based memristive device
Source: Front Comput Neurosci. 2022 Sep 14;16:1015945. doi: 10.3389/fncom.2022.1015945 (PMC9517377; doi:10.3389/fncom.2022.1015945)
Supplement: Supplementary file 1 [file Data_Sheet_1.DOCX]

Supplementary Material


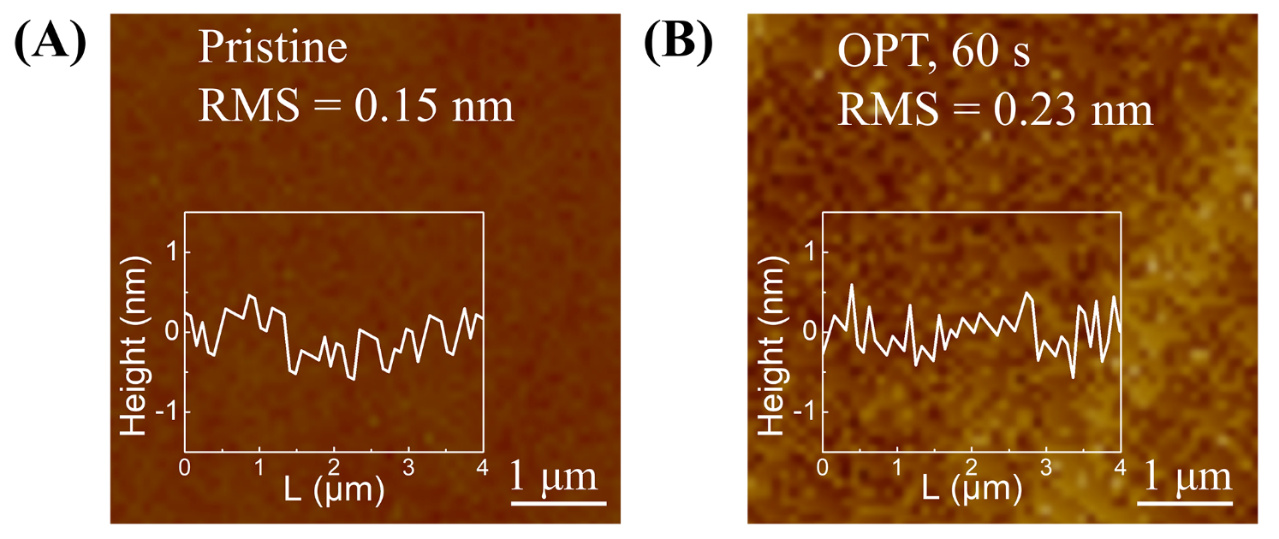


**Supplementary Figure 1.** AFM morphology of **(A)** pristine WS_2_ and **(B)** after oxygen plasma treatment for 60 s. The root-mean-square (RMS) roughness values calculated form the three maps are also presented. The insets in (A) and (B) are the representative height line scans.

**
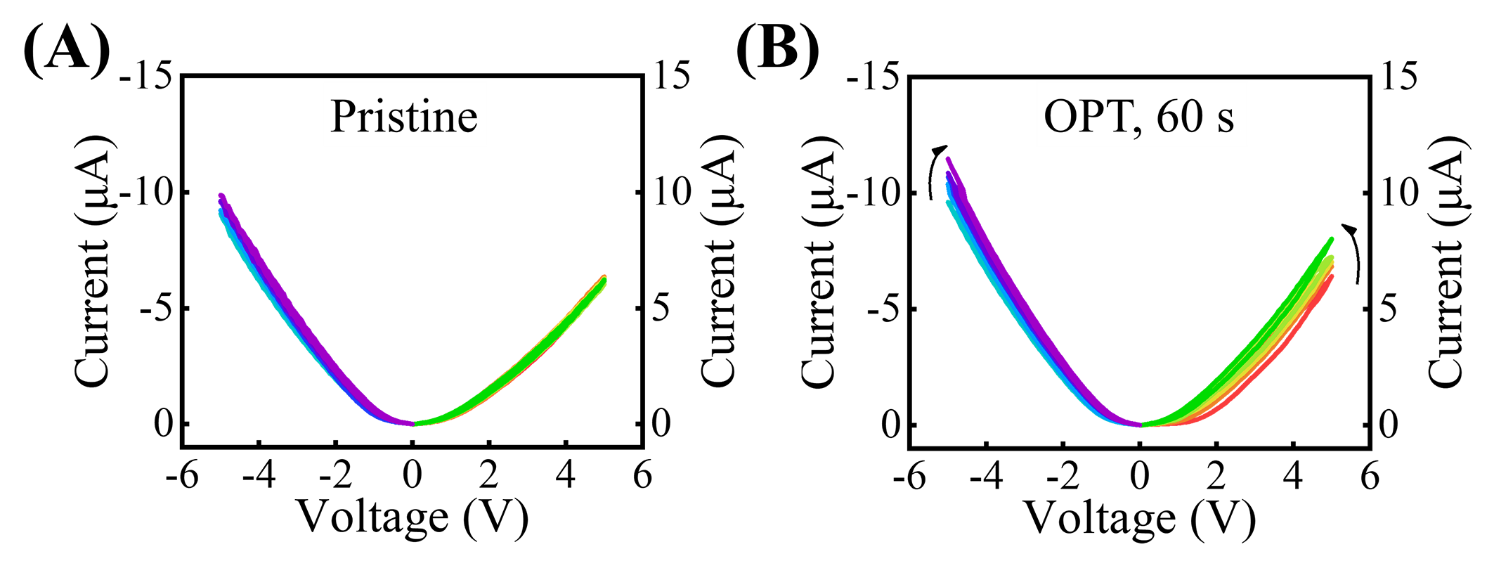
**

**Supplementary Figure 2.** Typical I-V curves of the memristive device based on (A) pristine WS_2_ and (B) WS_2_ after oxygen plasma treatment for 60 s.


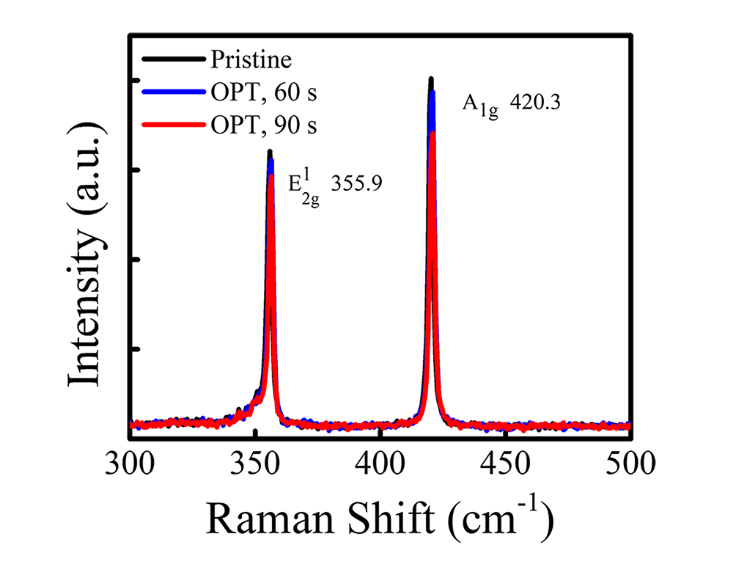


**Supplementary Figure 3.** Raman spectra of WS_2_ before and after oxygen plasma treatment for 60 and 90 s. There are two main peaks: One is a very sharp A_1g_ mode at high wavenumber of ~420 cm^-1^, representing the out-of-plane vibration; the other one is a broad $\text{E}_{\text{2g}}^{\text{1}}$ mode at lower wavenumber of ~356 cm^-1^, representing the in-plane vibration. No apparent Raman peak shift is detected, suggesting the WS_2_ crystal structure was unchanged after O_2_ plasma treatment.
